# A novel approach for protein subcellular location prediction using amino acid exposure

**DOI:** 10.1186/1471-2105-14-342

**Published:** 2013-11-28

**Authors:** Arvind Singh Mer, Miguel A Andrade-Navarro

**Affiliations:** 1 Computational Biology and Data Mining, Max Delbrück Center for Molecular Medicine, Robert-Rössle-Str. 10, Berlin 13125, Germany

## Abstract

**Background:**

Proteins perform their functions in associated cellular locations. Therefore, the study of protein function can be facilitated by predictions of protein location. Protein location can be predicted either from the sequence of a protein alone by identification of targeting peptide sequences and motifs, or by homology to proteins of known location. A third approach, which is complementary, exploits the differences in amino acid composition of proteins associated to different cellular locations, and can be useful if motif and homology information are missing. Here we expand this approach taking into account amino acid composition at different levels of amino acid exposure.

**Results:**

Our method has two stages. For stage one, we trained multiple Support Vector Machines (SVMs) to score eukaryotic protein sequences for membership to each of three categories: nuclear, cytoplasmic and extracellular, plus extra category nucleocytoplasmic, accounting for the fact that a large number of proteins shuttles between those two locations. In stage two we use an artificial neural network (ANN) to propose a category from the scores given to the four locations in stage one. The method reaches an accuracy of 68% when using as input 3D-derived values of amino acid exposure. Calibration of the method using predicted values of amino acid exposure allows classifying proteins without 3D-information with an accuracy of 62% and discerning proteins in different locations even if they shared high levels of identity.

**Conclusions:**

In this study we explored the relationship between residue exposure and protein subcellular location. We developed a new algorithm for subcellular location prediction that uses residue exposure signatures. Our algorithm uses a novel approach to address the multiclass classification problem. The algorithm is implemented as web server 'NYCE’ and can be accessed at http://cbdm.mdc-berlin.de/~amer/nyce.

## Background

The cell is a three-dimensional space separated into different compartments. These cellular compartments have different function and physicochemical environment. The cell’s functional machinery - proteins - need to be present at specific cellular compartments so that cells can function properly. Wrong localization of proteins may lead to disease and cell death [1]. Therefore, subcellular location is a key-feature in the functional characterization of proteins [2].

Currently, most protein sequences in databases are the result of translation of hypothetical transcripts derived from genomic sequencing data [3]. Therefore computational prediction of protein features from their sequence is often used for designing strategies for experimental characterization of proteins and is also important for genome annotation and drug target identification [4,5].

In particular, the computational prediction of subcellular location from protein sequence information has been attempted mainly using three approaches. One approach tries to identify motifs recognized by the sorting proteins and receptors of the protein transport machinery to move protein products from the cytosol to other subcellular locations [6]. This approach is limited by our knowledge of these signals; absence of detection of known motifs cannot be used to imply that a protein remains in the cytosol. A second approach uses sequence homology to proteins of experimentally verified localization under the assumption that similar proteins end up at similar subcellular locations [7,8]. While this is true in general terms, there are many known exceptions for this rule (e.g. the proteins of the Lsg1 family of GTPases [9] or locations taken as known might be predicted or incorrect.

A third approach uses the amino acid composition of the protein as a proxy for location based on the hypothesis that the physicochemical properties of the residues of a protein must be somehow coupled to the physicochemical properties of the environment where the protein performs its function; therefore the differences between environments will be imprinted in the protein amino acid composition [10,11]. This approach has the advantage that it can be applied to any set of compartments and proteins, provided one has enough data.

These three approaches have their strengths and disadvantages. A targeting signal prediction is, in principle, more reliable than a predicted location based on a close protein ortholog (or on a protein domain), which is itself better than location predicted on the basis of protein composition alone. However the existence of many proteins without known signals, known predicted domains associated to protein locations, or without homology to proteins of experimentally verified protein location, leaves room to make the prediction of protein location from composition alone a relevant objective.

While composition-based methods of prediction of location have not been extremely successful [12], we believe that these can be improved by using amino acid exposure. We previously studied how amino acid exposure influences the amino acid composition of proteins in different compartments and inferred that using this property should improve location prediction [13]. The rationale was that differently exposed residues have different evolutionary pressures to mutate towards specific amino acid types whose side chains have physicochemical properties that agree to the subcellular location where the protein performs its major activity. Since the publication of this previous work, much data on protein structures and experimentally verified protein locations have been deposited in public databases. Here, we present a novel analysis of the relation between protein amino acid exposure, residue type and subcellular location, which takes advantage of recent experimental data and methods for pattern-based classification and prediction of protein amino acid exposure.

As in [13], we will focus here on eukaryotic proteins and three locations: nuclear, cytoplasmic and extracellular. In addition, we will consider the necessity of introducing a fourth class and demonstrate that this can be predicted: proteins of nucleocytoplasmic localization. This class is not generally taken into account by methods of prediction of location, despite the fact that a large number of proteins are known to shuttle between nucleus and cytoplasm and perform functions in both compartments [14,15]. Failure to consider this abundant class might lead to mediocre performance in subcellular location prediction [16].

Therefore, in this manuscript we present a hybrid method that uses a support vector machine (SVM) and an artificial neural network (ANN), trained on proteins of known location and structure for the prediction of the four locations mentioned above: nuclear (N), nucleocytoplasmic (Y), cytoplasmic (C) and extracellular (E). Study of the training set and of the ranges of exposure with better prediction performance gave us insight into the relationship between amino acid exposure and environment, showing that predicting class Y improves the general prediction performance, but also suggesting unexpectedly that buried residues carry location information that is different from the information carried by exposed residues.

Our method was adapted for use on sequences of unknown structure by using predicted amino acid exposure values with reasonable performance. Application of the method to pairs of homologous proteins with different experimentally known location (e.g. two homologous proteins where one is localized to the nucleus and the other to the cytoplasm) indicated that the method can find the appropriate location in cases where methods using homology would make a wrong inference. Finally, we implemented the method as a web tool accessible at http://cbdm.mdc-berlin.de/~amer/nyce.

## Results and discussion

We developed an algorithm for protein location prediction that uses amino acid type and exposure to predict protein location. Our method benefits from the fact that there is evolutionary pressure for the selection of mutations that result in protein residues with side chains that have characteristic physicochemical properties according to the exposure of the residue and to the subcellular location of the protein. Our method does not use protein homology and accordingly can distinguish homologous proteins with different subcellular locations.

**Figure 1:**
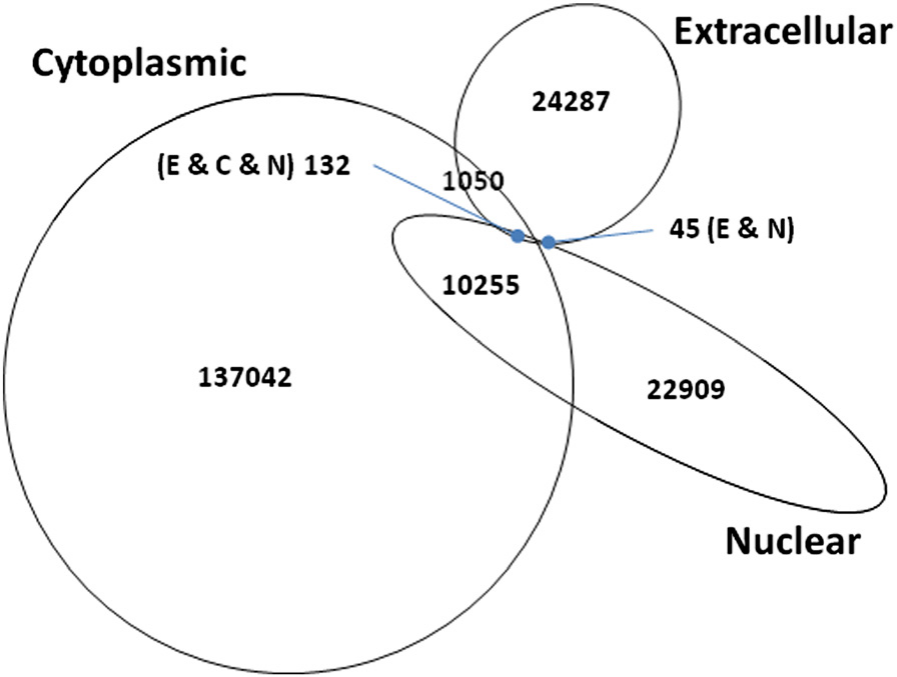
**Venn diagram of eukaryotic proteins exclusively found in three localization categories (selected from UniProt; see Methods for details).** A significant number of proteins are found both in the cytoplasm and in the nucleus.

To generate a training dataset we first selected proteins annotated to occur in three major locations: nuclear (N), cytoplasmic (C) and extracellular (E), and not in other locations (see Methods for details; Figure 1). Given the significant amount of proteins that shuttle between nucleus and cytoplasm (approximately one in three nuclear proteins) we considered an extra category (nucleocytoplasmic, Y). To obtain reliable information on amino acid exposure, we then selected proteins of known structure for each of these four categories (see Methods for details; Table 1). We obtained values of residue accessibility for all amino acids of the sequences in this dataset that were covered by 3D-structural information (see Methods).

**Table 1:** Number of proteins with PDB information

**Location**	**Proteins**
Nuclear	336
Nucleocytoplasmic	347
Cytoplasmic	543
Extracellular	132
Total	1,358

**Figure 2:**
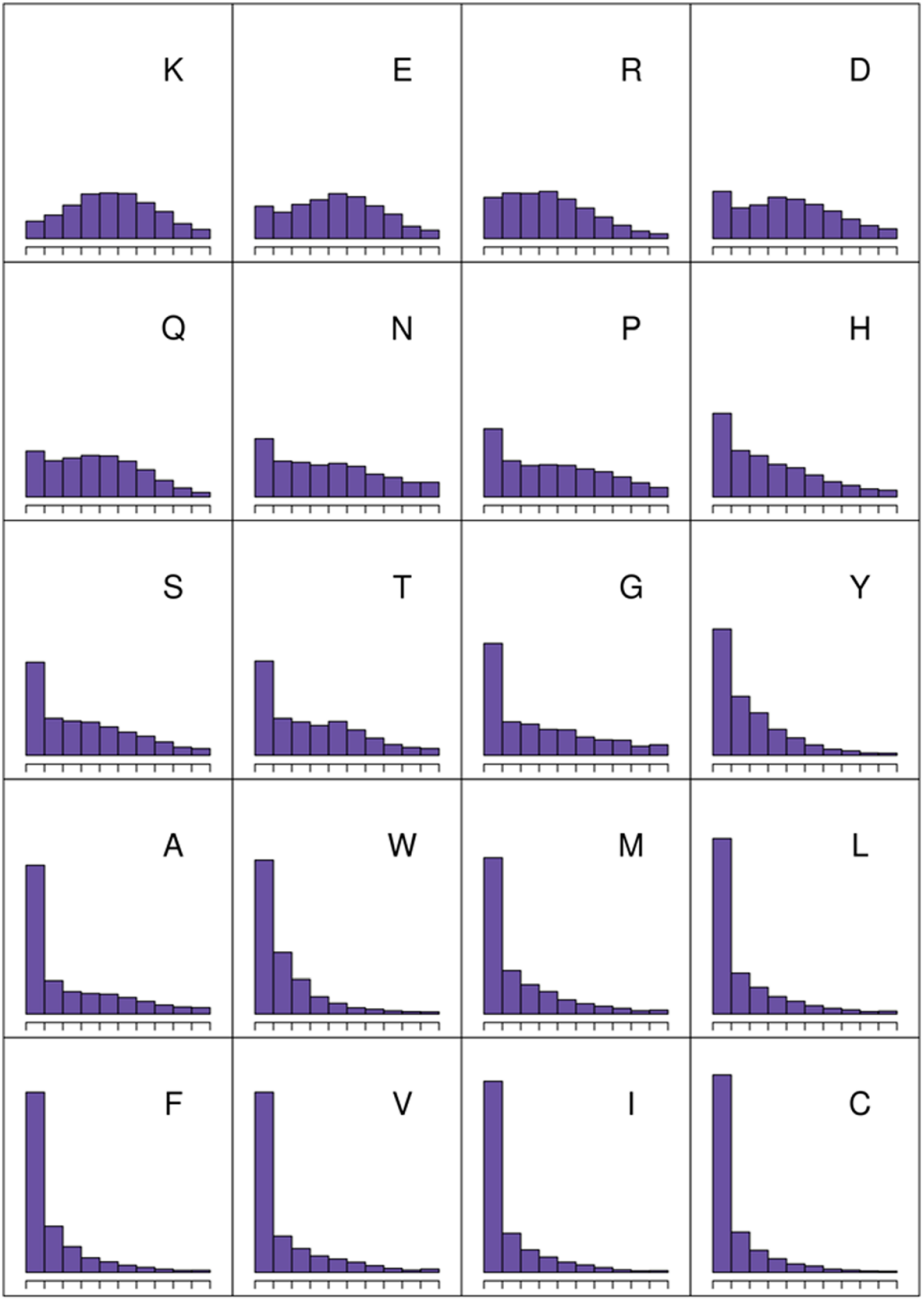
**Residue exposure frequency distributions (from buried to exposed) for each of the 20 amino acids in the proteins of known structure and experimentally verified location used to train the algorithm (Table **1**).**

**Figure 3:**
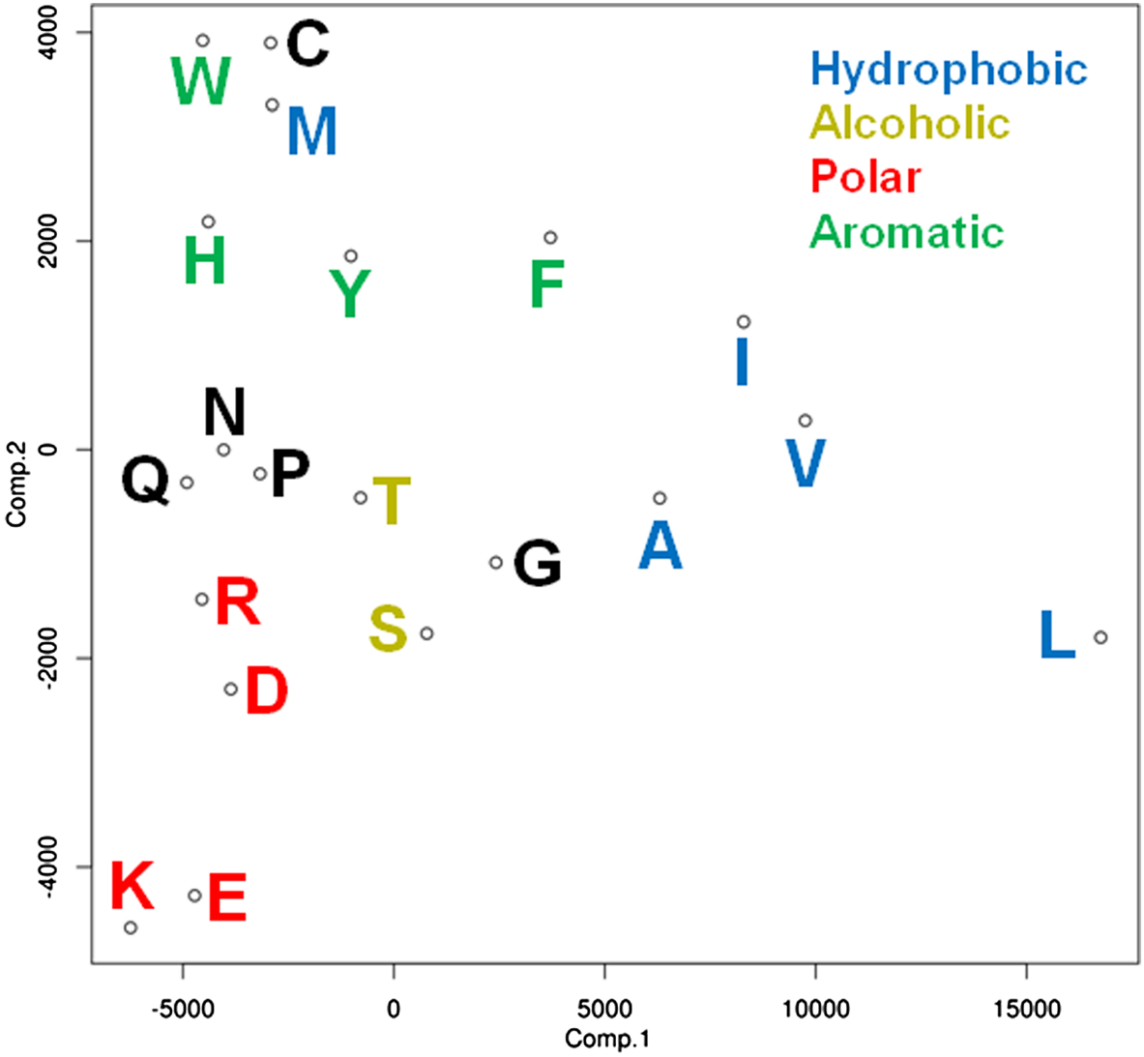
**Principal component analysis of the vectors of exposure of the 20 amino acids shown in Figure **2**.** Amino acids with similar properties appear close in the projection: polar residues like arginine **(R)**, aspartic acid **(D)**, glutamic acid **(E)** and lysine **(K)** group together. Same is true for alcoholic (threonine **(T)**, serine **(S)**) and aromatic (tryptophan **(W)**, histidine **(H)**, tyrosine **(Y)**, phenylalanine **(F)**) residues.

We then studied the distribution of exposure values for the 20 different amino acids. We observed that residues with side chains belonging to the same physicochemical property group show similar frequency distributions (Figure 2). For example the hydrophobic residues isoleucine (I), valine (V), leucine (L) and alanine (A) show very similar distributions with a very high frequency in the low accessibility region and fewer residues in the high relative accessibility region. Principal component analysis (PCA) of these data shows this more prominently (Figure 3).

**Figure 4:**
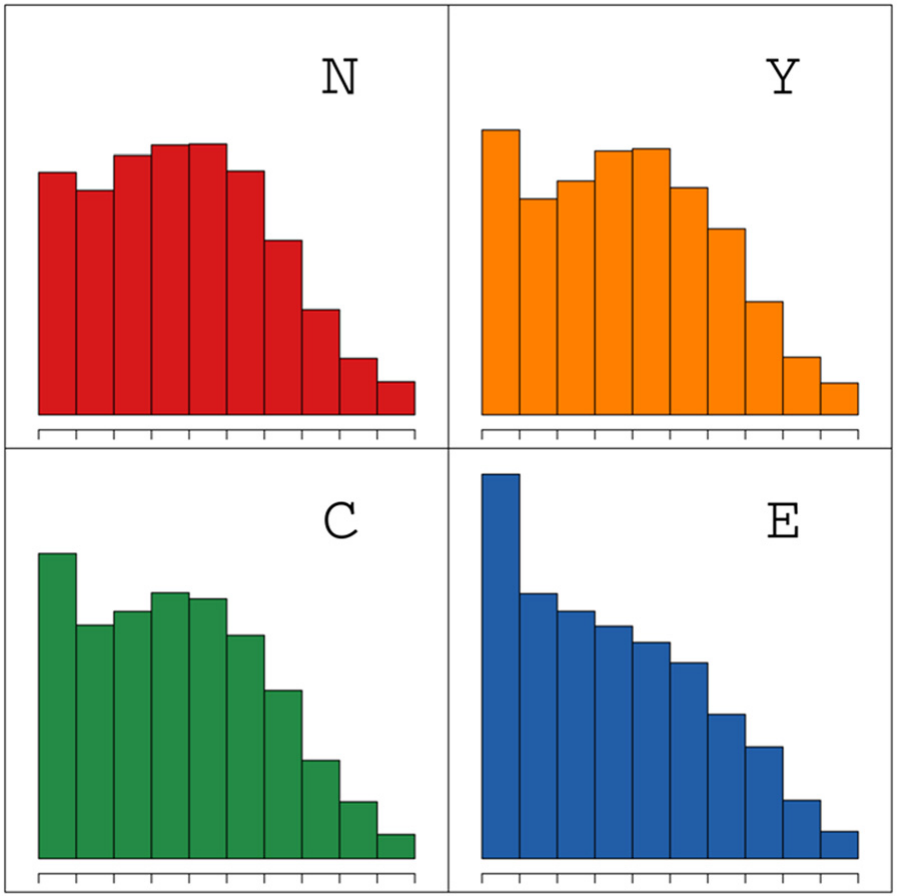
Distribution of values of exposure of glutamine (Q) in different location class proteins.

We then compared the distribution of exposure values for the 20 different amino acids in each of the four protein classes and observed variation for particular amino acids and protein locations (Additional file 1: Figures S1-S4). For example, when we compare the distribution of exposure values for glutamine (Q) in different location classes we can see that glutamines in extracellular proteins are more buried than in intracellular proteins (Figure 4). Conversely, cysteines in extracellular proteins have a distinct peak at high exposure values, which is absent from intracellular proteins (Additional file 1: Figures S1-S4). These differences imply that exposure values can be used to predict protein location.

### SVM classification using vectors of amino acid composition in selected ranges

Next we separated the values of amino acid exposure in six percentiles (1–6, from buried to exposed) and tested different vectors of amino acid composition for combinations of these six ranges. Initially we tried vectors with 20 components (one for each amino acid) describing the composition of residues found within a particular range of exposure values. For example, the range “1” composition vector for a protein would be defined by the distribution of amino acids of this protein with exposure values in the most buried category. The range “5 6” would be defined by the amino acids in the two most exposed categories. The range “1 2 3 4 5 6” would be the amino acid composition of the entire protein and so on.

**Figure 5:**
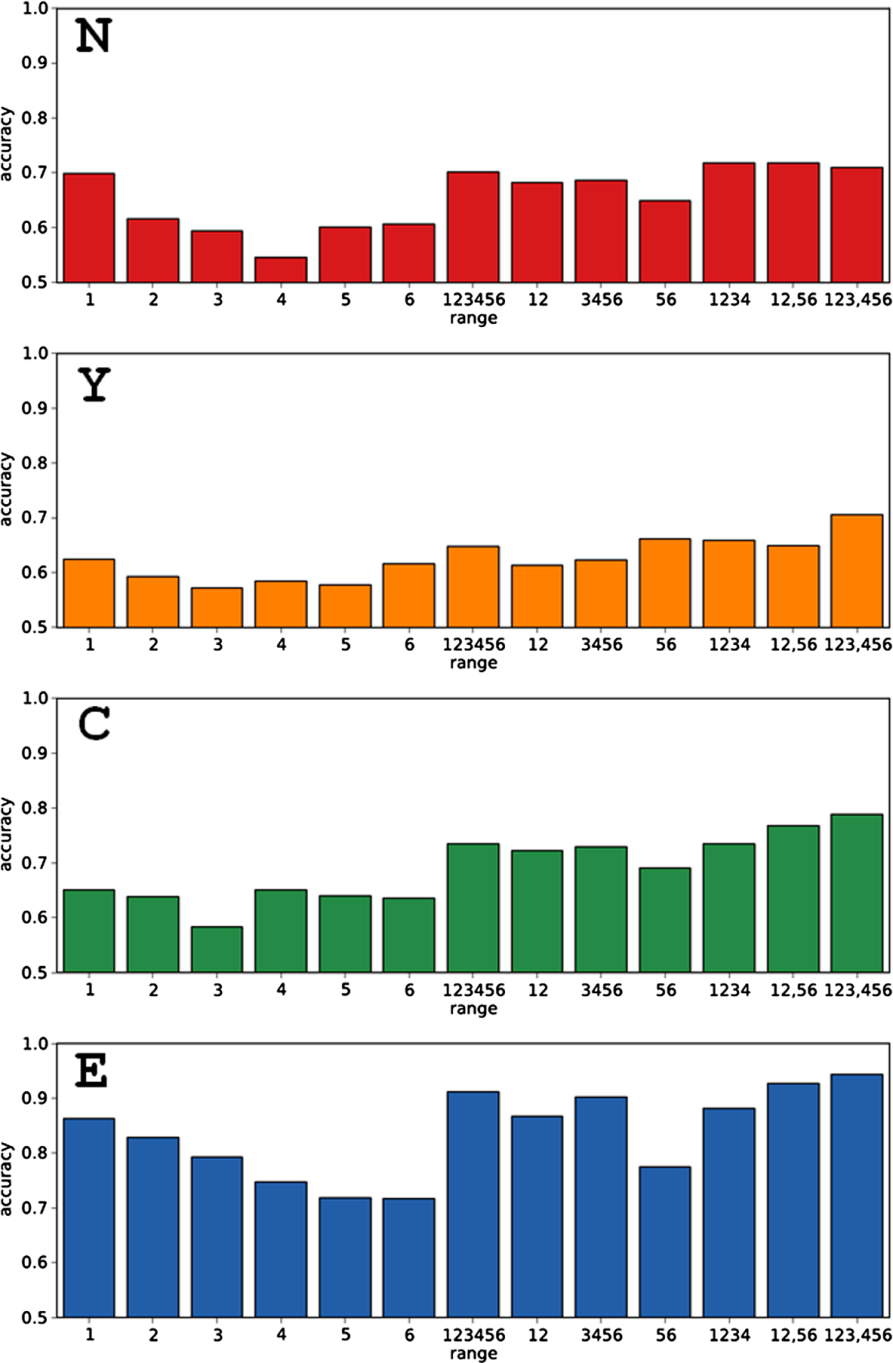
Accuracy of one-vs.-rest SVM classifications for nuclear (N), nucleocytoplasmic (Y), cytoplasmic (C) and extracellular (E) proteins using residues in different ranges of exposure (1–6, from buried to exposed; see text and Methods for details).

We then trained an SVM on such amino acid composition vectors for proteins from each of the four localization categories (see Methods for details). The accuracy of the classifier was distinctively better for extracellular proteins and worst for nucleocytoplasmic proteins (Figure 5). Interestingly, for nuclear proteins, and less so for nucleocytoplasmic and cytoplasmic proteins, the middle ranges of exposure (3 and 4) seem to contain less signal about the localization of the protein. For extracellular proteins, buried residues contain more information on the localization of the protein than exposed residues. In any case, the complete protein amino acid composition (full range: 1 2 3 4 5 6) was a better predictor than each of the six individual ranges, with composition from multiple ranges, e.g. (1 2), (3 4 5 6), close.

The bad performance of vectors of residues in smaller ranges may be due to the fact that we are dealing with proteins with an average size of 322 amino acids and the resulting range-specific amino acid composition vectors may be based on small numbers of amino acids. This effect is obviously reduced when the full range or a combination of ranges are used.

Since combined ranges seemed to perform next to full-range we wondered if combining these vectors could outperform full-range vectors. Therefore, we next tested SVM classifications using as training 40-component vectors that combined two different 20-component vectors. In particular, the 40-component vector combining the 20-component vector for residue composition in the three most buried categories with the 20-component vector for residue composition in the three most exposed categories (1 2 3, 4 5 6) provided on average better predictions than the full-range vector for the four location categories (Figure 5). Generally, this vector produced better results than other combinations excluding some ranges (e.g. (1 2, 5 6)) or using scrambled residue ranges (e.g. (1 3 5, 2 4 6), see below).

**Figure 6:**
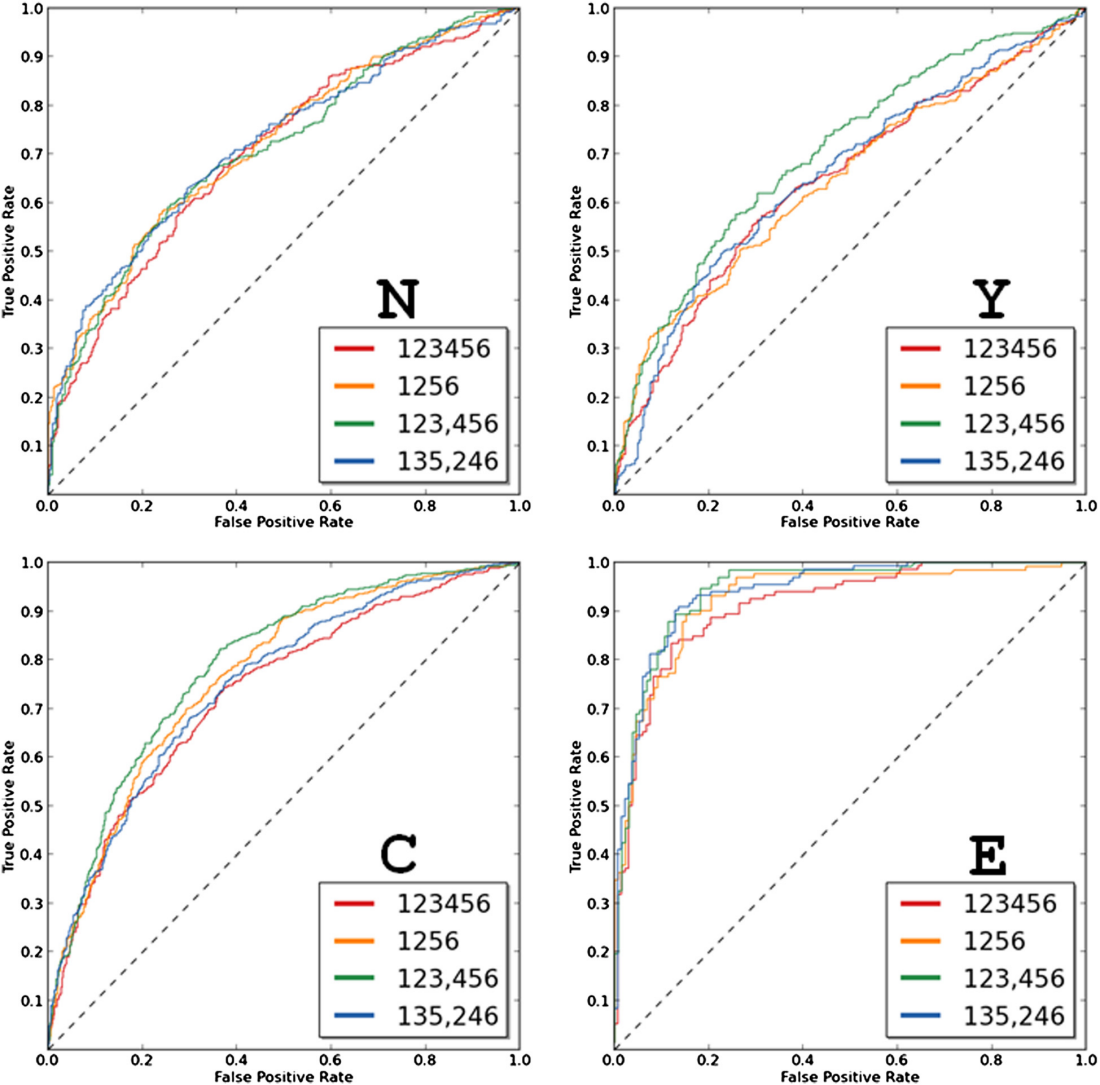
ROC curves of one-vs.-rest SVM classification for four location classes using composition vectors of residues in different ranges: 20-component vector based classification (ranges 123456 and 1256) and 40-component vector based classification (ranges 123,456 and 135,246).

Since from each one-vs.-rest SVM model we obtain a probability of being in a location class, it is possible to evaluate the accuracy of the model using a threshold for this probability. That is, we can compute the recall and precision of the predictions above various cut-offs of probability. The plot of these values as ROC (receiver operating characteristic) curves confirms that the extracellular class is predicted the best and that the 40-component vector (1 2 3, 4 5 6) provides better predictive power than full composition (Figure 6). To rule out the possibility that the superiority of the 40-component vector would be due to the higher amount of components, we tested a 40-component vector with scrambled ranges (1 3 5, 2 4 6), which performed poorly (Figure 6).

**Figure 7:**
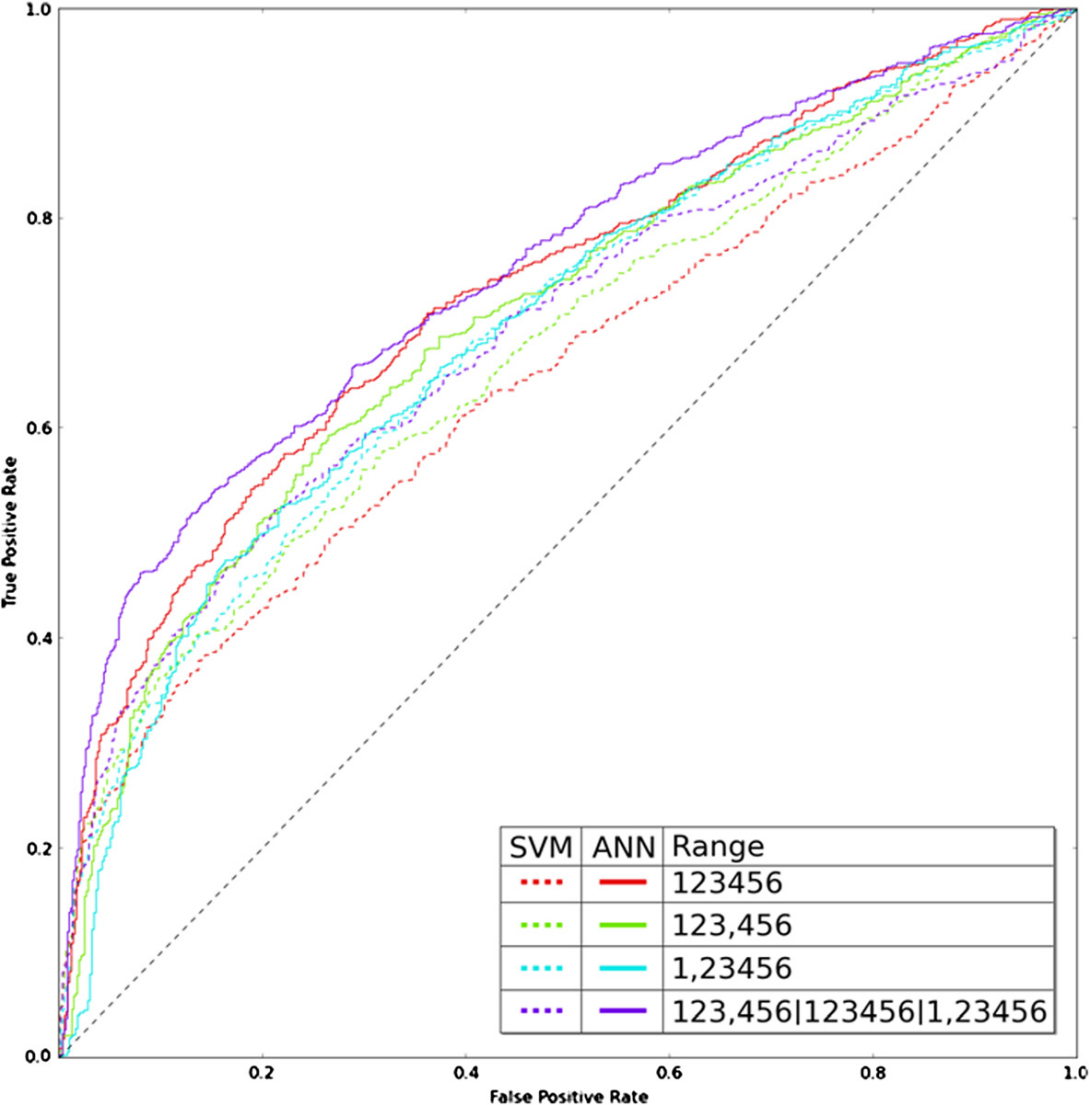
**ROC curves from SVM classifications (winner-takes-all strategy) and ANN classifications that use as input the SVM values.** For SVMs the ROC curves (dotted lines) were made by taking the best prediction from sets of SVMs (winner-takes-all strategy). Either best of four SVMs for each location category (red, green and cyan dotted curves indicating the different ranges used), or best of 12 SVMs (the combination of three SVM types is indicated with pipe signs indicating the vectors used; violet dotted curve). For ANNs the ROC curves (continuous lines) used just the ANN output. See text for details.

To combine multiple SVM predictions into a single one we applied a simple “winner-takes-all” strategy, that is, the prediction with best score is selected. ROC curves indicated that the 40-component vector (1, 2 3 4 5 6) performed best against other 40-component vectors (e.g. (1 2 3, 4 5 6)) or the full range 20-component vector (1 2 3 4 5 6) (Figure 7).

We then applied the “winner-takes-all” strategy to the three SVM sets mentioned above (that is, a set of 12 SVMs), but this did not improve performance significantly (dotted cyan curve in Figure 7).

### Combining class probabilities with an Artificial Neural Network

**Figure 8:**
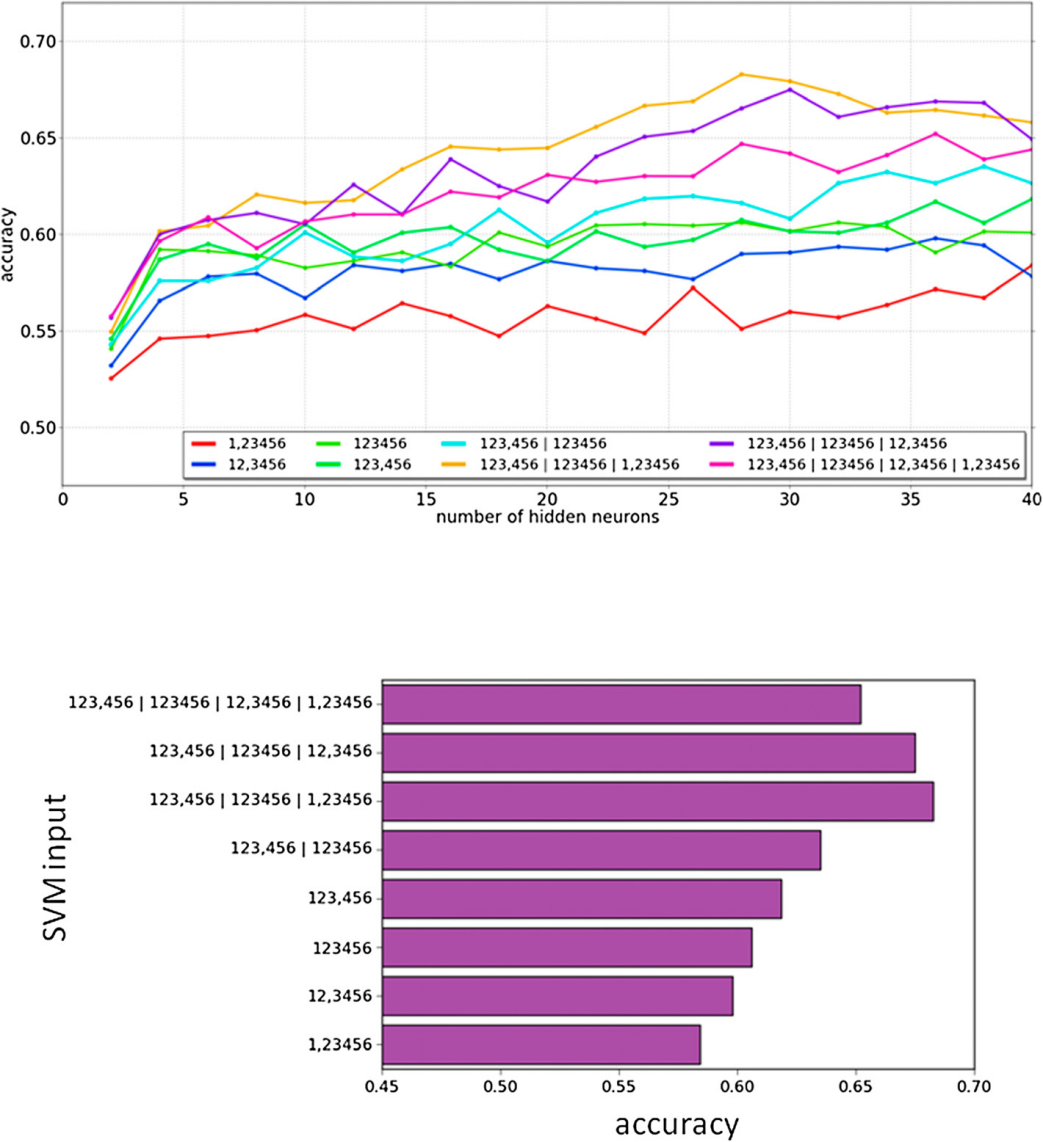
**Optimization of the artificial neural network (ANN).** (Top) ANNs were optimized using different numbers of hidden neurons and SVM types. (Bottom) Best accuracy value obtained. The legends indicate the type of SVM input used. SVM ranges and vectors (of 20 or 40 components) are indicated as in Figure 6. Use of multiple sets of SVMs are indicated by labels using “|” as separator. For example, the best accuracy value (0.68) was obtained using as input three sets of SVMs, two of them trained on 40-component vectors, and another trained on 20-component vectors.

We wondered if combining SVM scores for different localizations and ranges using an Artificial Neural Network (ANN), as opposed to just taking the best score prediction, could improve the accuracy of the method. To combine multiple SVM predictions for different locations we used an ANN with three layers: an input layer with one neuron for each of the one-vs.-rest SVMs used, a hidden layer, and an output layer with four neurons, one for each location class. The ANN was trained with SVM-calculated values and was required to produce an output of 1 for the correct class and 0 for the others (see Methods for details). The number of neurons in the hidden layer was optimized for maximum accuracy, as well as the type and number of SVMs using as input (see Methods for details; Figure 8). For example, we tried using four SVMs as input, one for each location class, but also tried using SVMs for two types of ranges (8 input neurons), three types of ranges (12 input neurons), and four ranges (16 input neurons).

The best result was obtained for 28 hidden-layer neurons and 12 input-layer neurons; the inputs were obtained from four SVMs using 40-component vectors for ranges 1 2 3 and 4 5 6, four SVMs using 40-component vectors for ranges 1 and 2 3 4 5 6, and four SVMs using 20-component vectors of full protein composition (accuracy 68%; see Figure 8). Increasing the number of SVMs used as input eventually decreased accuracy, probably due to over-training of the ANN. The final number of connections in the optimal ANN, (12 × 28) + (28 × 4) = 448, is well below the number of examples used for the training (1,358).

ROC curves for the ANN classifications indicate that they improve the predictions over the SVMs used as input, and confirm that the ANN selected performs the best (Figure 7). This combination of SVM inputs and ANN architecture was therefore selected for further work and finally for implementation as a public tool (see below).

### Predicting location of proteins without known structure

Our next goal is to apply the predictive architecture optimized above to protein sequences. Our method uses as input the composition of residues of a protein in six different ranges of exposure. However, generally, a given protein sequence has no 3D-information and therefore no known exposure values. Thus, we first need a method to provide predicted exposure values for the residues in the protein sequence whose localization has to be predicted.

To obtain predicted exposure values alone from sequence we have used a method that predicts exposure based on residue type and similarity to other sequences and that has high reliability (SABLE [17]; see Methods). The scoring system in SABLE is a scale of integer values from 0 (buried) to 9 (exposed). In principle, such a scale does not necessarily correspond directly to the scale of values of exposure that we obtained from proteins of known structure. After analysis of the distribution of SABLE values for the proteins of known structure used as training set, we equated SABLE scores 0 to 4 to our 3D-derived ranges 1 to 5, respectively, and the SABLE ranges of 5 and above (the less populated) to range 6, which was not perfect but approximated best the percentile distribution (see Methods, Table 2, and Additional file 1: Figure S5). The accuracy of the predictions with the optimal architecture SVM-ANN method was of 62%, which, as it could be expected, was lower than the value of 68% obtained when using the obviously more accurate 3D-derived values.

**Table 2:** Ranges of exposure used and their corresponding DSSP and SABLE values

**Range**	**DSSP**	**SABLE**
1	[0,0.01]	0
2	[0.01, 0.08]	1
3	[0.08, 0.21]	2
4	[0.21, 0.37]	3
5	[0.37, 0.57]	4
6	[0.57, 1.00]	[5, 9]

**Figure 9:**
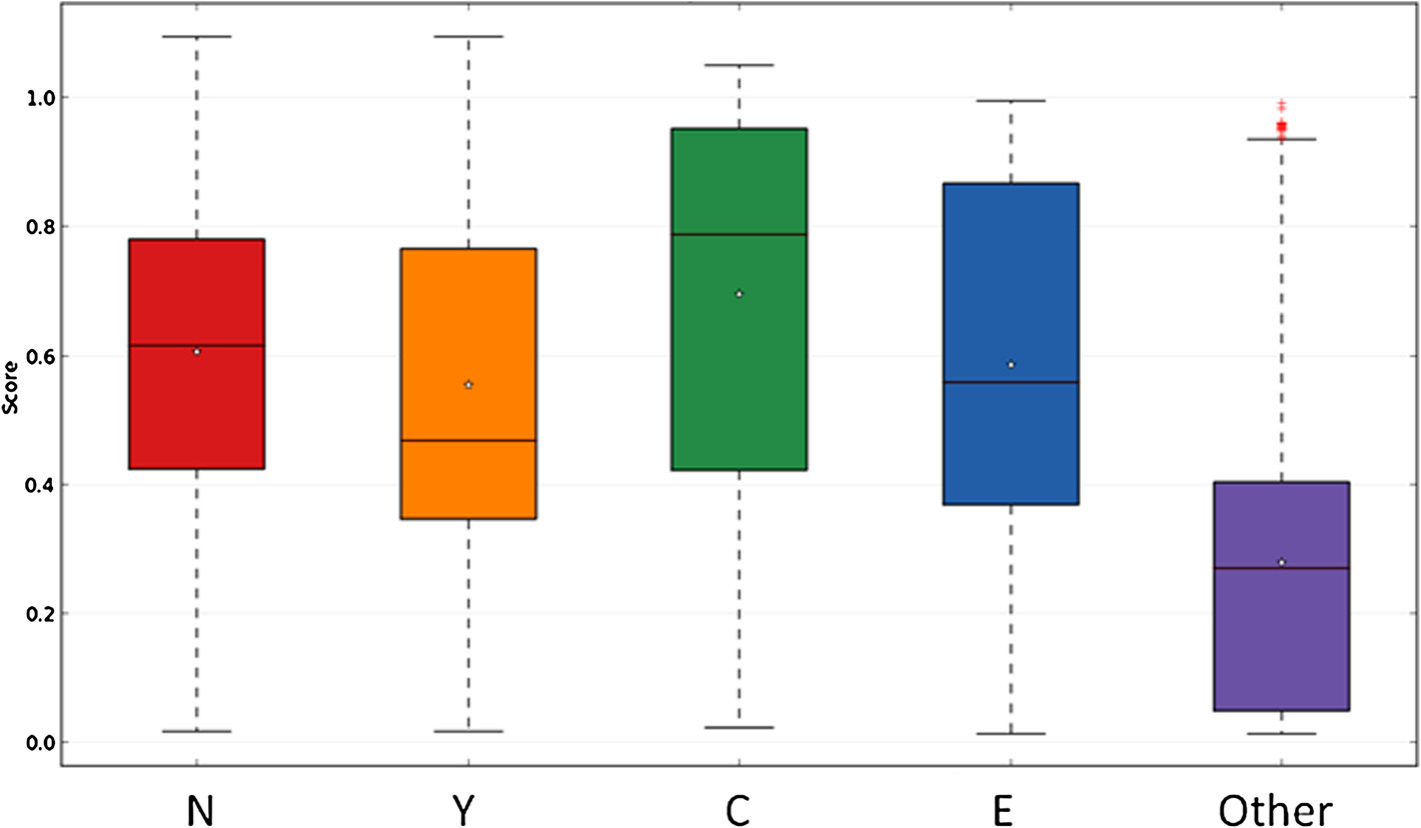
**Box-plot of the scores obtained in the classification of proteins from four locations (nuclear (N), nucleocytoplasmic (Y), cytoplasmic (C) or extracellular (E)) or from other locations (Other).** Proteins present in other locations received lower scores indicating that the method can discriminate between them.

Since the method was trained exclusively on proteins from four locations, we wondered if it would misclassify proteins present in other locations. To test this we ran the method on a set of 1358 eukaryotic proteins randomly selected from proteins with experimentally verified location but not assigned to nuclear, cytoplasmic or extracellular locations. We observed that more than 75% of these proteins not present in NYCE locations received scores below 0.4 (Figure 9). This threshold is used in the web tool to inform of the reliability of the predictions.

Predictions for 3320 human proteins without location annotation in UniProt are available as Additional file 2: Table S1.

### Predicting location of paralog protein pairs

As mentioned in the introduction, one of the main problems of protein subcellular location prediction methods based on homology is that there are very similar proteins that act in different subcellular locations. For example, the two well-known protein-tyrosine kinases BMX and FRK are cytoplasmic and nucleocytoplasmic, respectively; however they share 25% identity along over 60% of their sequences, mostly due to their two N-terminal domains (SH2-Protein kinase). Homology is therefore not necessarily the best criteria to assign location to proteins. To test that our method can evaluate proteins independently of their homology, we collected and analysed pairs of paralogs experimentally known to be in different NYCE locations such as the two tyrosine kinases mentioned above (see Methods). From a total of 64 such pairs our method predicted the same location for both proteins in only 27 cases, indicating that the method does not have a dependency on homology. The proper localization was correctly predicted for both sequences in 13 cases, which was significant compared to random tests where the pairs were assigned each of four localizations with equal probability (p-value 0.0015; Additional file 1: Figure S6).

Using the paralog pair data set, we compared NYCE to four other state-of-the-art subcellular location prediction tools: Yloc [18], Hum-mPLoc [19,20], SherLoc [21] and PSORT-II [22] (Table 3 and Additional file 3: Table S2; see Methods for details). For this set of protein pairs NYCE outperforms all other tools. All of these tools except PSORT-II use homology for location analysis.

**Table 3:** Performance of NYCE and other location prediction methods

**Tool**	**Number of correctly predicted proteins**	**Accuracy on proteins (in %)**	**Number of correctly predicted pairs**	**Accuracy on pairs (in %)**
NYCE	49	52.68	13	20.31
Yloc	42	45.16	3	4.68
Hum-mPLoc	35	37.63	3	4.68
SherLoc	40	43.01	0	0.00
PSORT II	37	39.78	10	15.62

## Conclusions

Our study demonstrated that the distribution of amino acids at different levels of exposure have signal about the location of proteins. Whereas exposed residues might have to adapt to the physicochemical properties of the environment and to interactions with particular macromolecular entities such as DNA, RNA, etc. [13], buried residues might also have location dependent roles; for example, extracellular proteins might have to have more stable cores to increase the stability of proteins exposed to conditions more variable than in intracellular regions [23]. While localization signals that guide protein sorting mechanisms are possibly the best predictor of a protein’s location, protein amino acid composition can be a useful predictor of location if such signals are absent or unknown.

On a technical note, our method illustrates how a multi-class problem can be approached by using a two-step approach where first SVMs of different types score class membership for each one of several classes and in a second step an artificial neural network (ANN) integrates the data and reassigns membership considering all scores from the SVMs. This approach could be especially useful for other classification tasks in cases like ours where the number of test cases is relatively small and limits the number of input and outputs of the ANN. For example, we could not have trained the ANN directly on the 20- and 40-component vectors used as input for the SVMs with the few hundreds of examples of eukaryotic proteins of known location and structure available. In this respect, the SVM step can be considered as a kind of data compression prior to the use of an ANN. A Bayesian approach might also be feasible for this second step.

We note that our method depends on the quality of the predicted exposure values. Although SABLE has already high accuracy in the prediction of protein amino acid exposure [17], further developments in this field could eventually be used to improve our predictions towards accuracy values close to those obtained when using 3D-derived values of amino acid exposure.

Applying our approach to other protein location prediction problems, for example, for prokaryotic proteins, or for additional eukaryotic locations, is certainly possible but results will depend on the amount and quality of experimental data on protein location and on the amount of signal for each location present in the sequences of experimentally verified location. Expanding our method will thus require careful selection of training datasets considering new taxonomic divisions and locations in a case by case basis. We expect that the development of novel techniques for high-throughput characterization of protein location might eventually facilitate such development.

## Methods

### Selection of proteins with known structure and known location

From the UniProtKB/Swiss-Prot database (release 2012_05) we obtained all eukaryotic protein-IDs. These protein-IDs were mapped to corresponding location information defined as UniProt terms in the Subcellular Location field of the UniProt record. For our analysis we removed all proteins annotated as located in any other than one of three locations: nuclear, cytoplasmic and extracellular. Interestingly there are a significant number of proteins annotated as nuclear as well as cytoplasmic (Figure 1). This led us to include another location class, 'Nucleocytoplasmic’, in our analysis. To improve the quality of data we removed the proteins whose location annotation is experimentally not yet verified (as indicated by the words “by similarity”, “probable” or “potential”). We also removed UniProt unreviewed records, and in the case of extracellular proteins, we removed glycosylated proteins (as indicated in the UniProt record) because glycosylation affects the surface properties of proteins [24].

These selected eukaryotic proteins were mapped to entries in the PDB database of protein structures. If multiple PDBs were available for a sequence we selected the PDB id corresponding to the longest sequence fragment. Since small proteins might not have enough residues to compute statistics on their exposed residues, we discarded sequences shorter than 150 amino acids. We ended up with a total of 336, 347, 543 and 132 proteins for nuclear, cytoplasmic, nucleocytoplasmic and extracellular locations, respectively, for a total of 1,358 proteins (Table 1).

### Computation of relative accessibility values for each residue

Each protein in the PDB has an associated entry in the DSSP database [25], which includes information on the exposure of each residue automatically inferred from the 3D structure. The values of relative accessibility to the solvent were calculated from the DSSP database for each of the residues of every selected protein. To calculate these values the ACC (accessibility) value (from DSSP) is normalized by the maximum residue accessibility for each of the 20 amino acids as defined by [26].

A value of 1 means high accessibility (that is, the residue is exposed to the solvent) and a value of zero means no accessibility (the residue is buried in the protein structure). Around 50% of all the residues of the proteins considered had a relative accessibility below 0.1, with 32% above 0.5 and only 10% about 0.9, but these values depend very much on the type of amino acid considered.

### Computation of amino acid composition vectors for proteins

The amino acid composition vector of a protein is a vector of 20 components, one for each amino acid. Each component *i* is the fraction of residues of type *i* in the protein. Therefore the sum of the components is equal to one. For particular calculations we compute the composition vector of residues in a given range of exposure. We also used 40-component vectors that combine two 20-component vectors.

We created six ranges of residue exposure values such that at every range there is almost an equal number of residues (Table 2). This allows us to compare and combine different ranges in terms of power for prediction of protein location.

### Extending a SVM for multiclass data classification

At the first step of classification we applied a support vector machine (SVM), a supervised machine learning method, using the software library LIBSVM, Version 3.11 [27]. SVMs are a binary classification algorithm, which we had to extend for our multiclass data. The multiclass (N-class) problem can be solved in two ways: one-vs.-one approach or one-vs.-rest approach. To solve N-class problems the one-vs.-one approach uses N*(N-1)/2 binary classification models and applies majority voting for a final decision. The one-vs.-rest approach uses N different models and a final decision is based on maximum probability (winner takes all). To decide between these classification strategies it is important to contemplate the nature of the classification problem [28]. Consider the case of a protein localized in the nucleus. Classifying this protein with the one-vs.-one approach will require 6 binary classification models out of which only 3 classifiers will have the option to classify the protein in the correct class, nuclear, while the other 3 classifiers will necessarily classify the protein in a category other than nuclear, therefore wrongly. Differently, the one-vs.-rest approach uses 4 classification models out of which 1 classifier will have the option to classify the protein in the nuclear class while the remaining 3 classifiers might correctly classify the protein in the 'rest’ category. Considering this fact we chose the one-vs.-rest approach for multiclass classification.

### Data balancing, training and optimization

Vectors of amino acid composition for our set of proteins of known structure and location using amino acids in different ranges of exposure were used as input data for LIBSVM.

Our dataset is highly unbalanced (Table 1). In an imbalanced dataset, where one class instance far outnumbers other class instances, SVMs perform poorly and can produce biased results. For instance, if a classifier classifies a data set where the class ratio is 3:1, a classifier can show 75% accuracy by classifying all data-points in the larger class. To overcome this problem we applied a data-balancing method. For each of the four location classes (N, Y, C and E) one was taken as positive and an equal sized negative dataset was created with members from the other three classes. When possible, the negative dataset contained the same amount of sequences for each of the 3 classes. When using C as positive set (543 sequences) there were not enough E proteins to be used as negatives (123 < 534 / 3 = 181). In this case we used all E proteins as negatives and took equally sized sets from Y and N proteins to complete the negative set (210 from each).

For each SVM training we performed a 10-fold cross validation. For this purpose the data is randomly divided into 10 sets. For each of the 10 cross validations one set is used as test data and the others are used as training data. To obtain an optimized SVM model we searched the parameter space of the SVM. The parameter values that produce best accuracy were recorded and used for the optimized model. As our training datasets are balanced it is safe to use accuracy as performance measure. Then the accuracy of the SVM was evaluated as the fraction of proteins in the test set correctly predicted. The average accuracy value is calculated from the 10-fold cross validation tests. Performance of different range vectors were compared using ROC (Receiver operating characteristic) curves.

An artificial neural network (ANN) was used for second level of classification. Our artificial neural network is a multilayer perceptron, which is trained using the back-propagation algorithm. The input layer of the network consists of 4, 8, 12 or 16 input neurons taking the probability values from one-vs.-rest SVM models for each of the four categories as input. The output layer of the neural network consists of 4 neurons, one for each location class. The number of neurons in the hidden layer was optimized by 10-fold cross-validation.

We tested different combinations of SVM models trained with different range vectors. The combination providing maximum accuracy was used as final model to implement the algorithm.

### Prediction of location for proteins without structural information

To predict the location of proteins without 3D-structure information, we computed residue exposure from sequence alone using the tool 'SABLE’ [29]. This tool predicts relative solvent accessibility of an amino acid residue on a scale from 0 to 9 with an approximate accuracy of 78%. As our final model is based on residues classified in six ranges of relative solvent accessibility values derived from DSSP, we needed to map the SABLE predicted solvent accessibility values to those 6 ranges. We did this by analysing the distribution of exposure values predicted by SABLE for the amino acids of the protein sequences with PDB information that we used to train the method. We then matched the 9 possible SABLE values to 6 ranges according to percentile distribution as well as possible (Table 2). These values are used to generate different range exposure vectors derived from SABLE values that are fed into the classification model. The algorithm finally scores a protein for its membership to the four location classes.

### Paralog selection

We applied our newly developed method to pairs of homologous human proteins obtained from the Eukaryotic Paralog Group Database [30]. We selected those pairs with sequences longer than 150 amino acids and with experimentally verified location information according to the UniProt record, exclusively nuclear, cytoplasmic, nucleocytoplasmic or extracellular. We then selected protein pairs with paired proteins in different subcellular locations. Using our method we predicted location for such protein pairs.

The same set of protein pairs was analysed using four other state-of-the-art subcellular location prediction tools: Yloc [18], Hum-mPLoc [19,20], SherLoc [21] and PSORT-II [22]. Yloc and Hum-mPLoc have the capacity to classify proteins into multiple locations. Thus, proteins classified as nuclear and cytoplasmic by these tools are equivalent to the nucleocytoplasmic class of NYCE. Although the tools SherLoc and PSORT-II do not consider nucleocytoplasmic as a separate class, they provide a score for each class. We utilized the nuclear and cytoplasmic class score from these tools to generate a nucleocytoplasmic class association. For this purpose we applied a simple strategy that if the normalised nuclear + cytoplasmic score together is greater than 50% the protein is considered as nucleocytoplasmic. A protein pair is considered correctly predicted if both the proteins are classified in the accurate location.

## Competing interests

The authors declare that they have no competing interests.

## Authors’ contributions

AM performed all computations and designed and implemented the web tool. Both authors designed the study, evaluated the results, and wrote the manuscript. Both authors read and approved the final manuscript.

## Supplementary Material

Additional file 1: Figure S1 Residue exposure frequency distributions for the dataset of nuclear proteins. **Figure S2.** Residue exposure frequency distributions for the dataset of nucleocytoplasmic proteins. **Figure S3.** Residue exposure frequency distributions for the dataset of cytoplasmic proteins. **Figure S4.** Residue exposure frequency distributions for the dataset of extracellular proteins. **Figure S5.** Distributions of DSSP and SABLE scores and mapping to ranges. **Figure S6.** Assignment of location to pairs of paralogs is significantly better than random. The green line represents accuracy of our method versus the distribution of accuracies obtained from random simulations. In only 1499 cases out of 1e6 the result of the random test was better than our method (see text for details). Click here for file

Additional file 2: Table S1 Location predictions for 3320 human proteins without location annotation in UniProt. Click here for file

Additional file 3: Table S2 Results of the comparison of NYCE to other location prediction methods. Click here for file
