# Supplementary material for: A novel approach for protein subcellular location prediction using amino acid exposure
Source: BMC Bioinformatics. 2013 Nov 28;14:342. doi: 10.1186/1471-2105-14-342 (PMC4219330; doi:10.1186/1471-2105-14-342)
Supplement: Additional file 1: Figure S1 — Residue exposure frequency distributions for the dataset of nuclear proteins. Figure S2. Residue exposure frequency distributions for the dataset of nucleocytoplasmic proteins. Figure S3. Residue exposure frequency distributions for the dataset of cytoplasmic proteins. Figure S4. Residue exposure frequency distributions for the dataset of extracellular proteins. Figure S5. Distributions of DSSP and SABLE scores and mapping to ranges. Figure S6. Assignment of location to pairs of paralogs is significantly better than random. The green line represents accuracy of our method versus the distribution of accuracies obtained from random simulations. In only 1499 cases out of 1e6 the result of the random test was better than our method (see text for details). [file 1471-2105-14-342-S1.pdf]

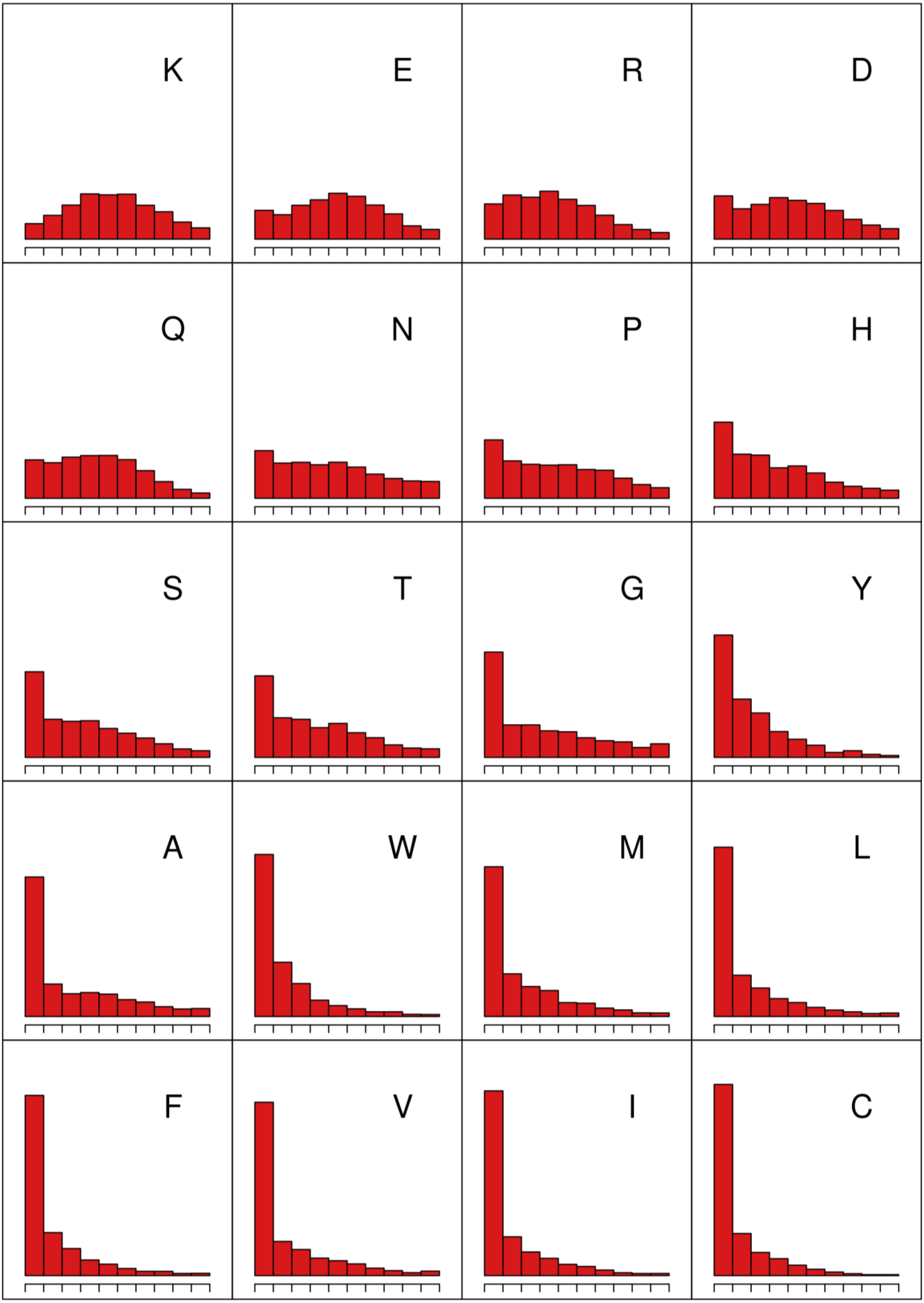

Figure S1- Residue exposure frequency distributions for the dataset of nuclear proteins

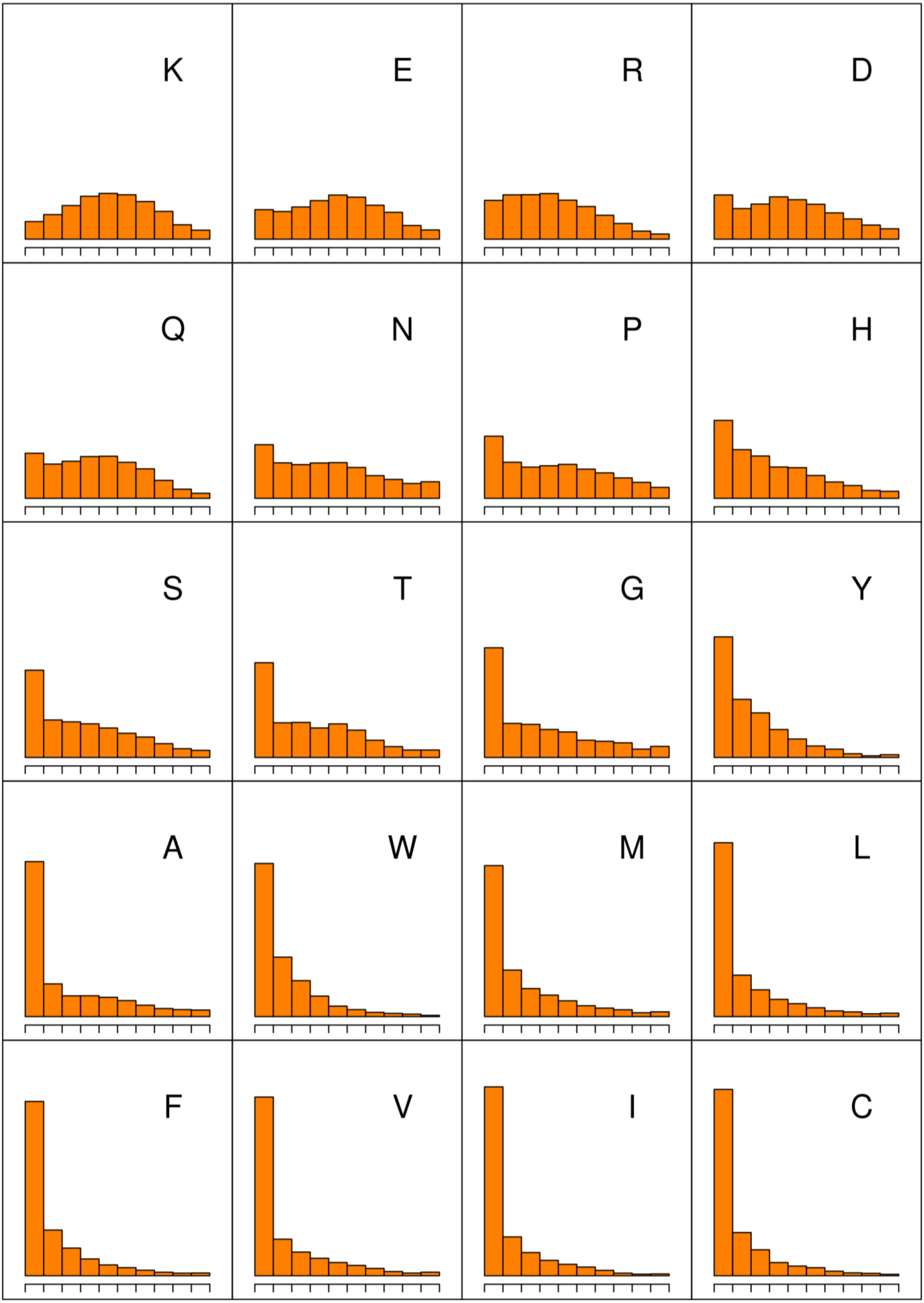

Figure S2- Residue exposure frequency distributions for the dataset of nucleocytoplasmic proteins.

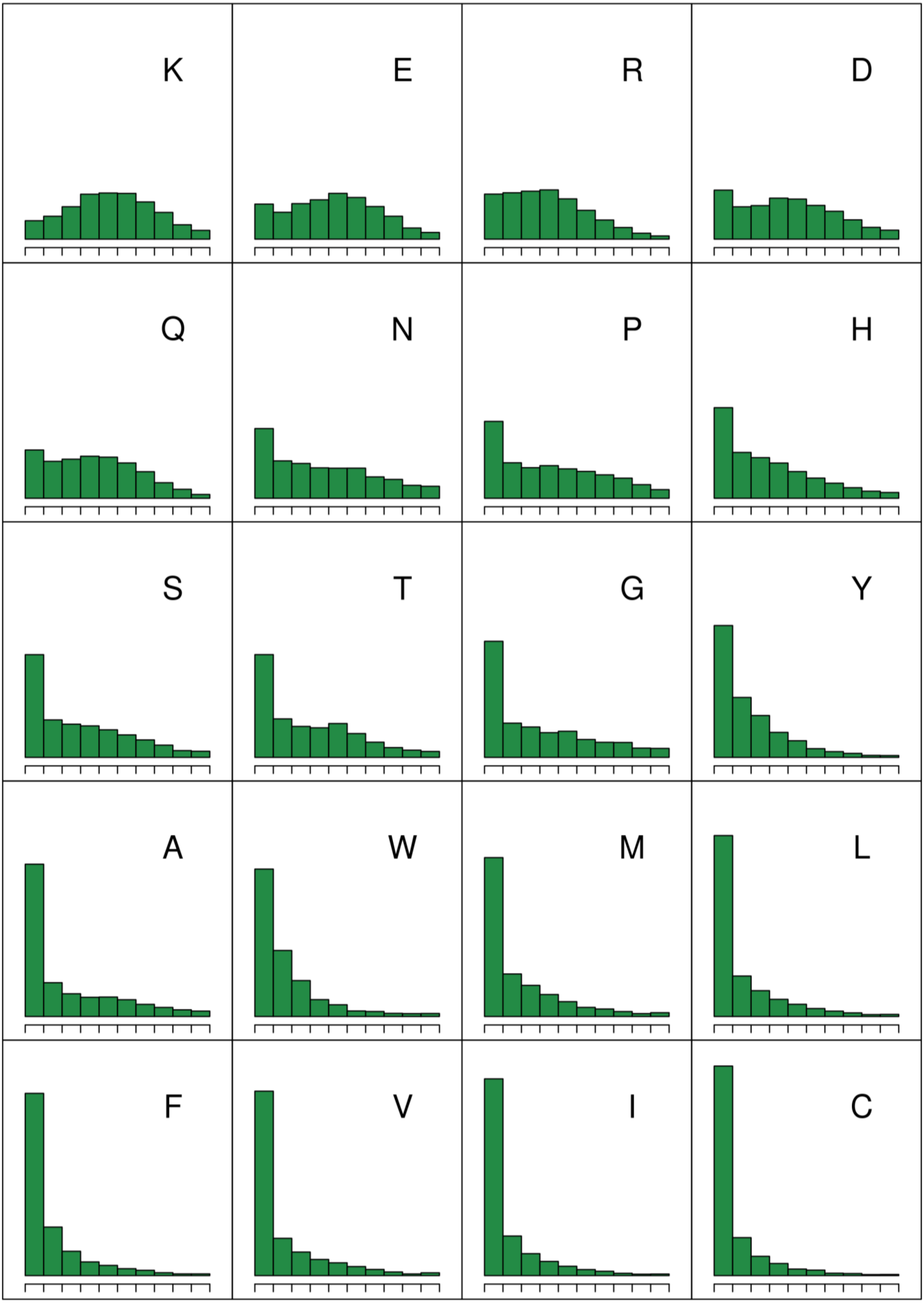

Figure S3. Residue exposure frequency distributions for the dataset of cytoplasmic proteins.

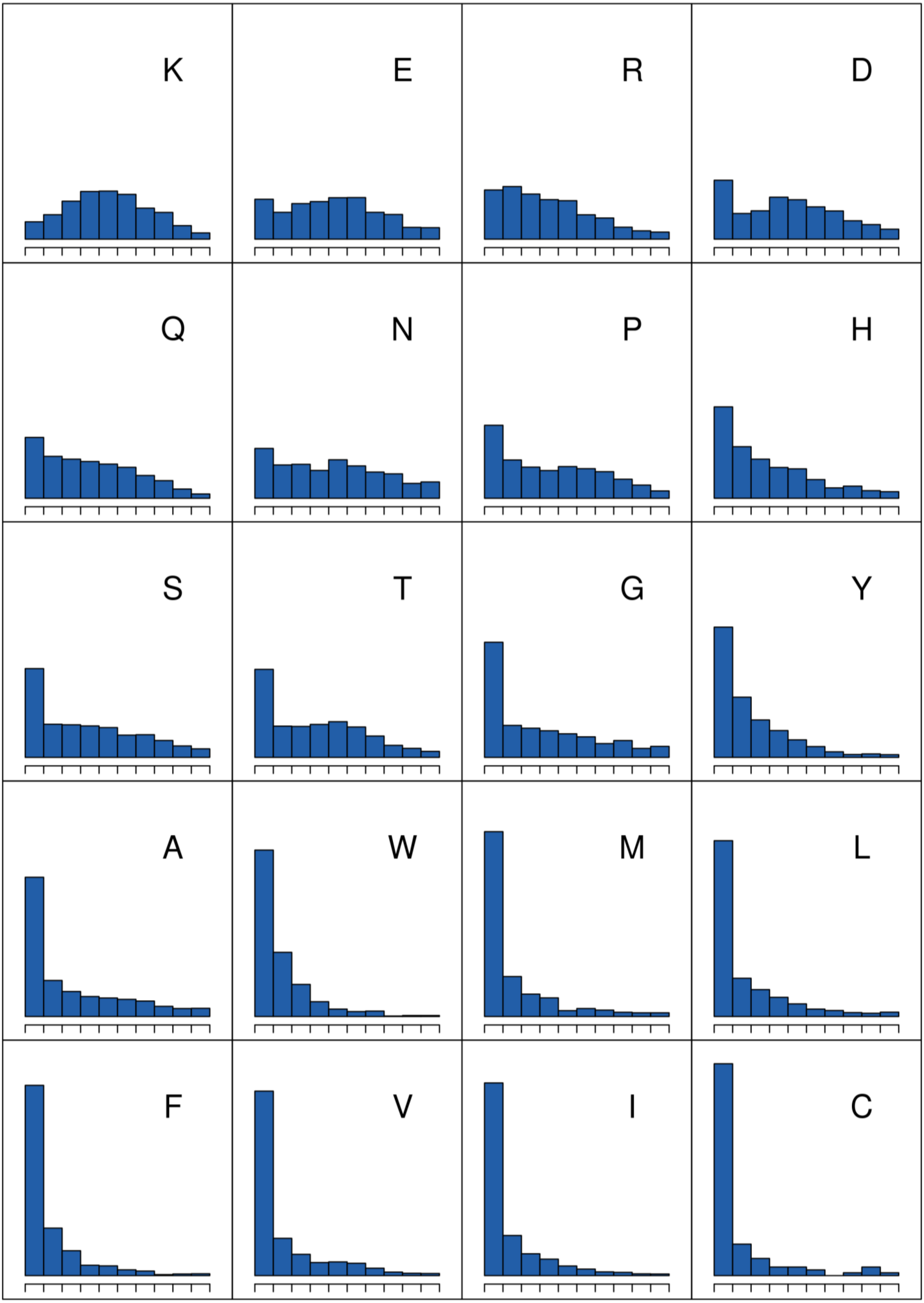

Figure S4. Residue exposure frequency distributions for the dataset of extracellular proteins

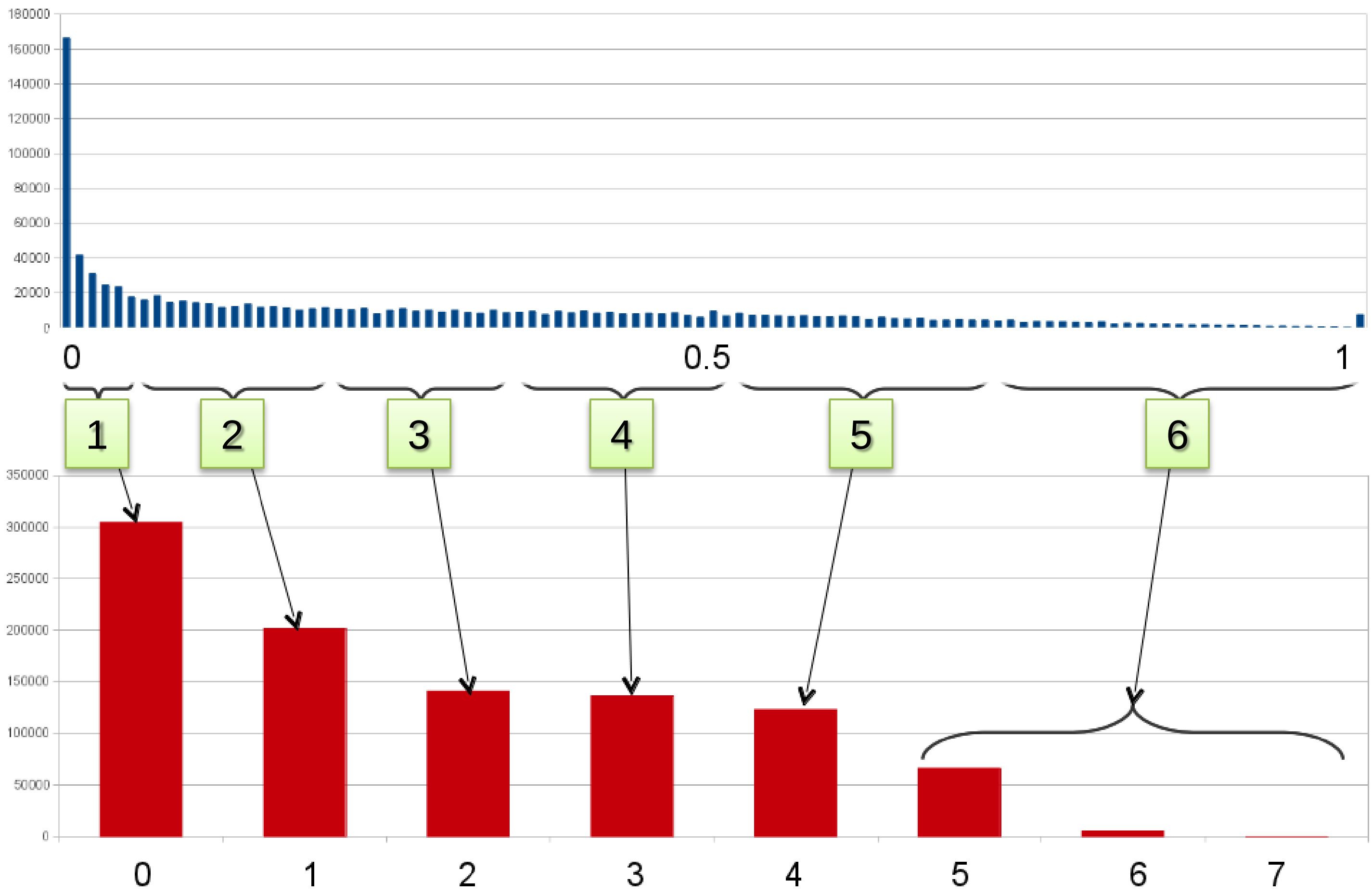

Figure S5. Distributions of DSSP and SABLE scores and mapping to ranges.

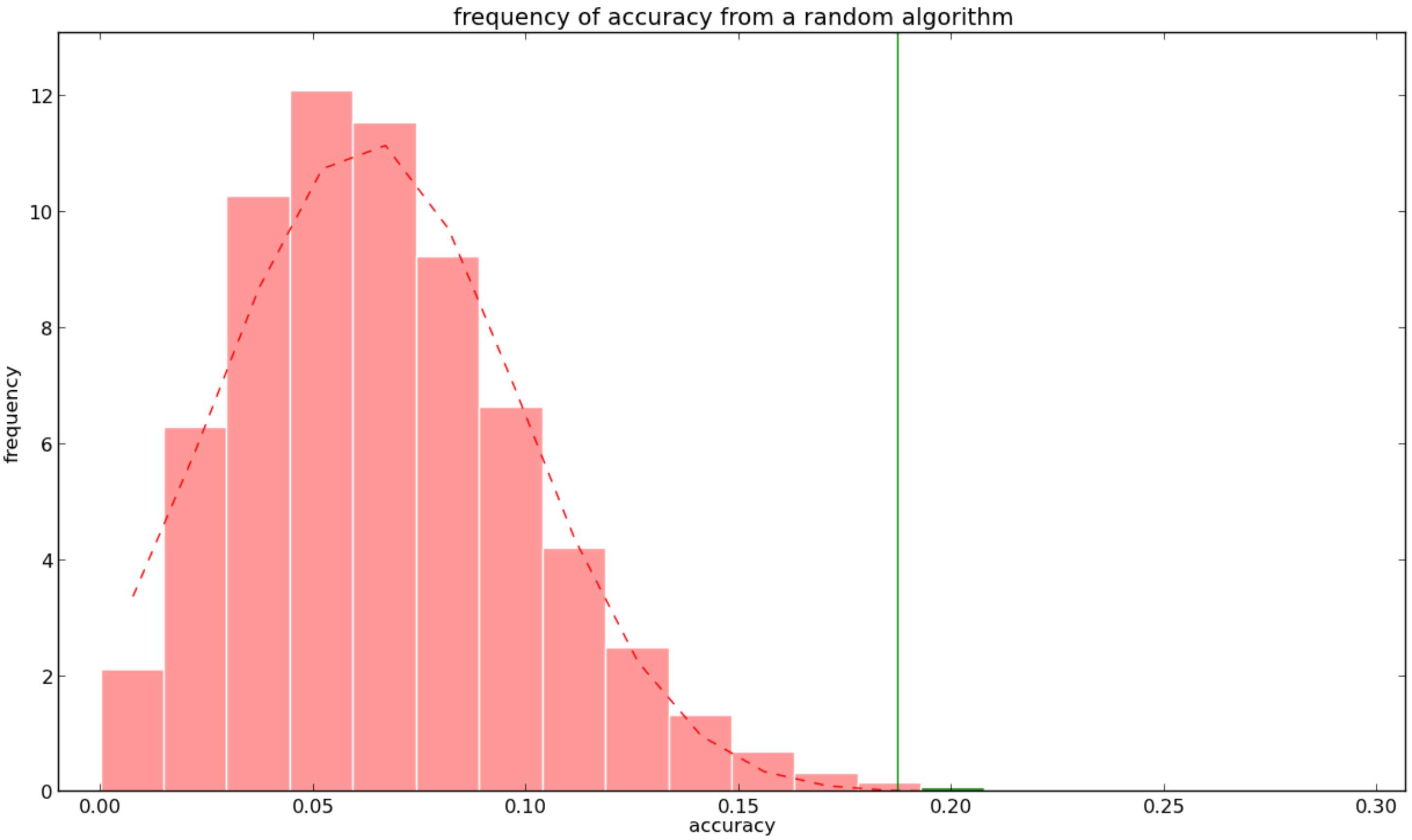

Figure S6. Assignment of location to pairs of paralogs is significantly better than random. The green line represents accuracy of our method versus the distribution of accuracies obtained from random simulations. In only 1499 cases out of 1e6 the result of the random test was better than our method (see text for details).
